# Characterization of the Antibiotic Compound No. 70 Produced by *Streptomyces* sp. IMV-70

**DOI:** 10.1100/2012/594231

**Published:** 2012-03-12

**Authors:** Lyudmila P. Trenozhnikova, Almagul K. Khasenova, Assya S. Balgimbaeva, Galina B. Fedorova, Genrikh S. Katrukha, Nina L. Tokareva, Boo H. Kwa, Azliyati Azizan

**Affiliations:** ^1^Institute of Microbiology and Virology, Ministry of Education and Science Committee, 103, Bogenbay batyr Street, Almaty, Kazakhstan; ^2^Research Institute of New Antibiotics, Russian Academy of Medical Sciences, Moscow, Russia; ^3^Global Health Department, College of Public Health, 13201 Bruce B. Downs Boulevard, Tampa, FL 33612, USA

## Abstract

We describe the actinomycete strain IMV-70 isolated from the soils of Kazakhstan, which produces potent antibiotics with high levels of antibacterial activity. After the research of its morphological, chemotaxonomic, and cultural characteristics, the strain with potential to be developed further as a novel class of antibiotics with chemotherapeutics potential was identified as *Streptomyces* sp. IMV-70. In the process of fermentation, the strain *Streptomyces* spp. IMV-70 produces the antibiotic no. 70, which was isolated from the culture broth by extraction with organic solvents. Antibiotic compound no. 70 was purified and separated into individual components by HPLC, TLC, and column chromatography methods. The main component of the compound is the antibiotic 70-A, which was found to be identical to the peptolide etamycin A. Two other antibiotics 70-B and 70-C have never been described and therefore are new antibiotics. The physical-chemical and biological characteristics of these preparations were described and further researched. Determination of the optimal growth conditions to cultivate actinomycete-producer strain IMV-70 and development of methods to isolate, purify, and accumulate preparations of the new antibiotic no. 70 enable us to research further the potential of this new class of antibiotics.

## 1. Introduction

In the recent decades, pathogenic microorganisms with resistance to common drugs have become increasingly widespread; some examples include methicillin-resistant [1–4] strains of *Staphylococcus aureus* (MRSA), vancomycin-resistant strains of *S. aureus* (MRCNS or “methicillin-resistant coagulase-negative staphylococci”), drug-resistant strains of *Streptococcus pneumoniae* (DRSP), and vancomycin-intermediate strains of *S. aureus* (VISA) [1–4]. MRSA have been viewed as one of the leading nosocomial pathogens [5–7]. In the last decade of the 20th century, there has been an unprecedented spread of MRSA on a global scale. According to the data of the National Nosocomial Infections Surveillance in the USA, in 1998, the proportion of MRSA in intensive therapy wards reached 50%. At the same time the rate of MRCNS occurrence rose to 70–80% without substantial variation by region. These developments lead to significant complications in treating infections and pose a serious challenge to modern medicine. The search for new natural antibiotics that overcome drug resistance of pathogenic microflora is a viable solution to this problem. Such research necessitates the systematic screening of producers of antibacterial antibiotics.

As a result of screening from Kazakhstan soils, six strains of actinomycetes that produce antibiotics were isolated. These strains produce antibiotics with a wide range of action that is highly active against gram-positive microorganisms. The new antibiotics were found to be highly active against clinical strains of MRSA and MRCNS that exhibited multiple associated resistances to the main groups of antibiotics which included *β*-lactams, macrolides, aminoglycosides, ftorchinolons, lincomycin, and chloramphenicol. Based on the initial study of its physical-chemical properties which includes analysis of activity against a series of clinical agents during *in vitro* experiments as well as acute toxicity in mice, the new antibiotic compound no. 70 was researched further for its therapeutic potential.

The actinomycete strain IMV-70, which produces antibiotic no. 70 that showed high levels of antibacterial activity, was isolated as a result of screening in the Institute of Microbiology and Virology of Ministry of Education and Science of Kazakhstan. This paper describes the research of the producer strain IMV-70 and the highly active antibiotic compound no. 70 which it produces. Preliminary results have been published earlier [8].

## 2. Material and Methods

### 2.1. Strains and Media

The following bacterial strains were used for testing antimicrobial activity: *Comamonas terrigena* ATCC 8461, *Bacillus subtilis* ATCC 6633, *Staphylococcus aureus* FDA 209 P, and clinical strains of methicillin-resistant staphylococci spp., isolated from the materials of the Central Clinical Hospital of the Medical Presidential Center of Kazakhstan. Identification of clinical strains and determination of their drug resistance were conducted on the automatic bacteriological analyzer “mini API” (bioMérieux, Marcy l'Etoile, France) with systems ID 32 STAPH and ATB STAPH 5. Strains of *Staphylococcus haemolyticus* NMR 1626, NMR 1742, *Staphylococcus epidermidis* NMR 351, NMR 793, *Streptococcus agalactiae* NMR 303, NMR 685, and NMR 1002, and *Enterococcus faecalis* NMR 425, NMR 118, NMR 670, and NMR 733 were isolated from urine; *S. haemolyticus *NMR 1642 and NMR 1656 from prostatic fluid; *S. agalactiae* NMR 782 and *Streptococcus oralis* NMR 1565 from respiratory tract specimen; *S. epidermidis* NMR 813 from vaginal smear; *S. epidermidis* NMR 1694 and *Enterococcus hirae* NMR 459 from wound matter; *S. haemolyticus* NMR 781 and *Staphylococcus lugdunensis* NMR 348 from nasal smear; *S. haemolyticus* NMR 713 from eye mucus; *Streptococcus pneumoniae* NMR 1150 from throat smear of infected patients.

Bacterial species (staphylococci, streptococci, and enterococci) for the tests were cultivated overnight at 37°C on Columbia blood agar (AG Medical Company, India). The antibiotic-producing bacterium, actinomycete strain IMV-70, was isolated from a mud sample, collected in 2000 from the lake Kyzylkol in Southern Kazakhstan. The IMV-70 strain was characterized based on the data from the research of its morphological, chemotaxonomic, and cultural characteristics by means of standard methodologies [9–13]. In order to study the morphology of its reproductive structures, the strain was grown on oat agar at 28°C. The type of spore chains was determined on the 10th day of growth. Study of morphological characteristics of the strain IMV-70 was conducted using trinocular microscope Leica DMLS with the digital video camera Leica DC 300F. The strain was deposited in the collection of the Scientific Center of the Russian Federation Research Institute for Genetics and Selection of Industrial Microorganisms; the collection number is VKPM As-1792.

### 2.2. Cultivation of the IMV-70 Strain and Extraction of Secondary Metabolites

In order to obtain spores, the IMV-70 strain was grown on mineral agar no. 1 and organic agar no. 2 for 10 days at 28°C as previously described [10]. The composition of mineral agar no. 1 used was (g/L) soluble starch 20.0; K_2_HPO_4_ 0.5; MgSO_4_ 0.5; NaCl 0.5; KNO_3_ 1.0; FeSO_4_ 0.01; agar 20.0; pH 7.2–7.4. The composition of organic agar no. 2 used was (%): Hottinger's broth 3 mL; peptone 5.0; glucose 10.0; NaCl 5.0; agar 20.0; pH 7.0–7.2. The vegetative inoculum was grown in 750 mL Erlenmeyer flasks containing 100 mL of medium and incubated at 28°C for 48 hours on a rotary shaker at 180–200 rpm. The amount of inoculum used for inoculation of the cultivation medium equaled 1% (spore suspension = 10^9^/mL). The composition of cultivation medium used was (g/L) corn extract (weight when dry) 5.0; (NH)_2_SO_4_ 3.5; NaCl 5.0; CaCO_3_ 5.0; insoluble starch 15.0; glucose 10.0. The amount of inoculum for inoculation of fermentation medium equaled 3% (vegetative mycelium). The compositions of the fermentation media used were (A) glucose 1.0; soy flour 1.0; NaCl 0.5; CaCO_3_ 0.25, and (B) yeast extract 5.0; glucose 20.0; peptone 10.0; CaCO_3_ 2.0; pH 7.3. Before sterilization, the pH value was raised to between 7.4 and 7.6 using 0.1 N NaOH.

**Figure 1:**
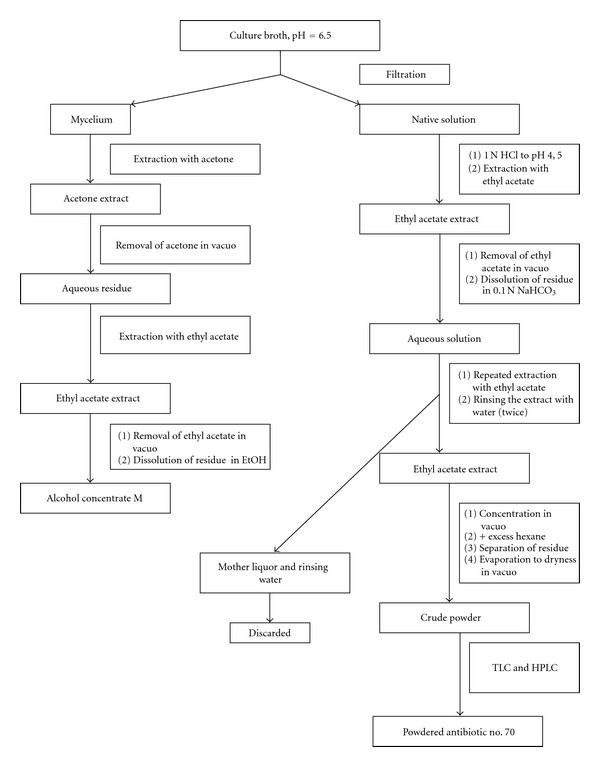
Flowchart for isolation of antibiotic no. 70. Antibiotic no. 70 was isolated from culture broth of the producer IMV-70 by extraction methods. The flowchart for the extraction that produces the powder preparation of compound no. 70 is shown in Figure 1. The antibiotic no. 70 was isolated from culture broth by extraction with ethyl acetate, was subsequently purified from the inactive lipid fraction, and was extracted from the concentrated solution with hexane.

Biosynthesis of the antibiotics was conducted in 750 mL Erlenmeyer flasks containing 100 mL of medium at 28°C for 96 hours on a rotary shaker. Antibiotic no. 70 was isolated from culture broth of the producer IMV-70 by extraction methods. The flowchart for this extraction is shown in Figure 1.

Analytical and preparative chromatography was carried out by the TLC method on the DC-Alufolien Kieselgel 60 “Merck” (Germany) and Silufol (Czech Republic) plates in a T1 solvent system, comprised of chloroform-benzene-methanol (30 : 20 : 7) and T2: methanol-water (96 : 4). Purification and separation of the antibiotic compound into individual components were also conducted by the method of column chromatography on the Kieselgel 60 “Merck” (Germany) silica gel. Suspension of silica gel in hexane was loaded onto a column, and then the dry preparation mixed with silica gel was added. The column was developed sequentially using different organic solvents: hexane, chloroform, mixture of chloroform and methanol (90 : 10), and methanol. Eluates from the column were analyzed by the TLC method on the DC-Alufolien Kieselgel 60 “Merck” (Germany) plates, with subsequent development of chromatograms by the method of bioautography with *Bacillus subtilis *ATCC 6633 as the test organism. Active heterogeneous fractions were united, dried in vacuo at 37°C, and dissolved in the minimal amount of ethanol. The antibiotic activity was analyzed by the paper-disk agar diffusion method (diameter of disks-6 mm) with *Bacillus subtilis *ATCC 6633 (= RIA 445) and *Staphylococcus aureus *INA 00761 (MRSA) as test organisms. HPLC analysis was conducted on liquid chromatographer “Knauer” (Germany) with automated data-processing system and spectrophotometric detector K = 2501; this was a stainless steel column (4 × 150 mm), filled with sorbent “Diaspher-110-C16, 5 mcm” and “Chromasil A-100 C-18, 5 mcm” (BioChimMac, Moscow). The chromatographic conditions used were as follows: flow rate, 1 mL/min; detection at wave lengths 260 and 300 nm; room temperature; loop, 20 *μ*L. Preparative isolation of components of the antibiotic compound A-70 was carried out through elution with a mixture of acetonitrile and water; the content of acetonitrile was gradually increased from 10 to 60%. The UV ranges were measured on spectrophotometer Shimadzu 1601C (Japan). The mass ranges were measured on Ultraflex 2 Tof/Tof by Bruker Daltonics (Germany) in the positive ion mode, matrix 2,5 dihydroxybenzoic acid. 

Analytical and semipreparative electrophoresis on paper were conducted on V-shaped Durruma unit with the following electrolytes: E1: 2 AcOH; pH 2.4; 550 V; 2 hr. and E2: 85% HCOOH-AcOH-H_2_O (28 : 20 : 52); pH 1.1; 250 V; 2-3 hr. Bioautography was conducted by methods described in the literature [14]. *Bacillus subtilis *ATCC 6633 (= RIA 445) and *Staphylococcus aureus *FDA 209P (MSSA) and INA 00761 (MRSA) were used as test organisms. 

### 2.3. Determination of the Antibacterial Activity by the Agar Diffusion Method

Sterile disks (AG Medical Company, India) containing 10 *μ*g/mL of crude powder of antibiotic no. 70 in EtOH were placed on fresh plates of the Mueller-Hinton agar seeded with bacterial suspensions at a cell density of 5 × 10^5^ CFU/mL of overnight cultures of the test microorganisms. The Mueller-Hinton agar was poured into the standard 80 mm double dishes, at a volume of 20 mL per dish. The diameters of the zones of inhibition of growth around the disks were measured after incubation periods of 18 h at 37°C.

The minimal inhibitory concentrations (MICs) were determined by the broth dilution method by using the Mueller-Hinton broth (MHB). The tubes containing 2 mL of serial twofold dilutions of each antimicrobial agent per well were inoculated with 0.2 mL of a bacterial suspension to yield a cell density of 5 × 10^6^ CFU/mL. MHB alone (tube with 2 mL) had no effect and was used as control. The tubes were incubated for 48 h, and visible growth was recorded after 18, 24, and 48 h of incubation. The MIC recorded was the lowest antibiotic concentration that completely prevented visible growth after incubation at 37°C for 18 h.

## 3. Results

The producer strain IMV-70, related to the genus* Streptomyces,* was isolated in the year 2000, at the Institute of Microbiology and Virology MES RK. The optimal media for biosynthesis of the antibiotic A-70 were found to be the organic media with yeast and corn extracts, as well as soy, pea, and oat flour. The antibiotic was formed at the highest rate in the medium with yeast extract and peptone on the 4th–7th day of fermentation and will be described in greater detail below.

### 3.1. Producer Strain IMV-70

The producer strain IMV-70 forms aerial mycelium with a diameter of hyphae of approximately 0.3–0.6 *μ*M (data not shown). Spore chains shaped like hooks, loops, and spirals were observed (RA, retinaculum-apertum). Occasionally, long straight chains were also observed (RF, rectus-flexibilis). The strain IMV-70 forms long cylindrical spore chains that contain more than 10 spores per chain and have a smooth surface (data not shown). Cultural characteristics were studied after two weeks of cultivation. The strain preserves stable cultural characteristics during cultivation when grown on different media, which included mineral agar, organic agar, sucrose-nitrate agar, inorganic salts-starch agar (ISP No. 4), glucose-asparagine agar (ISP No. 5), oatmeal agar (ISP No. 3), tyrosine agar (ISP No. 7), and peptone-yeast agar with iron (ISP No. 6). On the researched media, the strain forms a soluble pigment in the color range from light brown to brown. Melanin was not formed on the media ISP No. 6 and 7. Physiological analyses of strain characteristics revealed that strain IMV-70 utilizes the majority of carbon sources. The strain has tyrosinase, amylolytic and weak gelatinase activity, and peptonized milk. The taxonomic study identified the strain as *Streptomyces sp.* IMV-70. The tests of fermentation conditions indicated that the media components had significant influence on the formation of this antibiotic component. The optimal media for biosynthesis of the antibiotic no. 70 were the organic media with yeast and corn extract, as well as with soy, pea, and oat flour. The maximum antibiotic activity of culture broth develops on the 4th–7th day of fermentation. Addition of CoCl_2_ and ZnSO_4_ in the concentration of 0.0005 g/L to the media significantly stimulates antibiotic formation. The antibiotic no. 70 was isolated from culture broth by extraction with ethyl acetate and was subsequently purified from the inactive lipid fraction and extracted from the concentrated solution with hexane (Figure 1).

### 3.2. Antimicrobial Activity

Antibiotic no. 70 was found to be highly active against the following test organisms: *Comamonas terrigena* ATCC 8461, *Bacillus subtilis* ATCC 6633, and *Staphylococcus aureus* FDA 209 P. Minimum inhibitory concentration is within the range of 0.25–0.05 *μ*g/mL (Table 1(a)). The agar diffusion method was used for determining the activity of antibiotic no. 70 against various clinical strains of staphylococci, streptococci, and enterococci (Table 1(b)). The antibiotic no. 70 highest activity was against staphylococci and streptococci, whereas its activity against enterococci was weaker.

The highest activity of antibiotic no. 70 was recorded against clinical cocci strains: MRSA and MRCNS with different types of drug resistance (data not shown). The MIC for the collected group of clinical pathogen was within the range of 0.1–0.025 *μ*g/mL. To summarize the antimicrobial activity, we found in this study that the antibiotic no. 70 was active against gram-positive and gram-negative test organisms tested: *Bacillus anthracis *(I Tsenkovsky's vaccine*), Bacillus anthracoides, Staphylococcus aureus 209 P, Pasteurella multocida, Mycobacterium citreum*, and *Klebsiella pneumoniae 444*. The minimal inhibiting concentration of the powdered preparations of the antibiotic no. 70 with different maximum values of absorption in UV light was as follows: the preparation with *λ*_max_ 260 nm was 0.5–0.05 *μ*g/mL while the preparation with *λ*_max_ 300 nm was 0.25–0.05 *μ*g/mL. The antibiotic no. 70 was also found to be active against virulent cultures of *Listeria monocytogenes, Erysipelothrix rhusiopathiae*, and* Y. pestis*. Its activity is especially high against clinical cocci strains: staphylococci, streptococci, micrococci, enterococci, and aerococci with different types of drug resistance. Activity of the antibiotic no. 70 was studied against clinical MRS-strains with resistance to macrolides (erythromycin), aminoglycosides (gentamicin), lincosamides (lyncomycin), and ftorchinolons (ciprofloxacin, ofloxacin, and levofloxacin). In the tests with staphylococci strains, the minimal inhibiting concentration of the powdered preparation A-70 with *λ*_max_ 260 nm equaled 0.4–0.1 *μ*g/mL; the preparation with *λ*_max_ 300 nm required 0.1–0.025 *μ*g/mL.

According to the Government Standard 12.1.007-76 (requirements for detergents and domestic chemical products; Principles on Good Laboratory Practice implemented as National Standard in 2010), the powdered preparations of the antibiotic no. 70 were classified as belonging to the 4th class of toxicity : slightly toxic substances (for white mice LD_50_ at inoculation into stomach > 5000 mg/kg) (data not shown). Toxic, embryotoxic, and local irritating action of the antibiotic no. 70 was not discovered. The experiments with MRSA-induced sepsis in white mice determined that antibiotic no. 70 had distinctive medical effect for 90% of the infected mice and increased their lifespan (data not shown). During the autopsy, only isolated abscesses were discovered in the internal organs of the mice, treated with the preparation.

### 3.3. Chemical Identification

**Figure 2:**
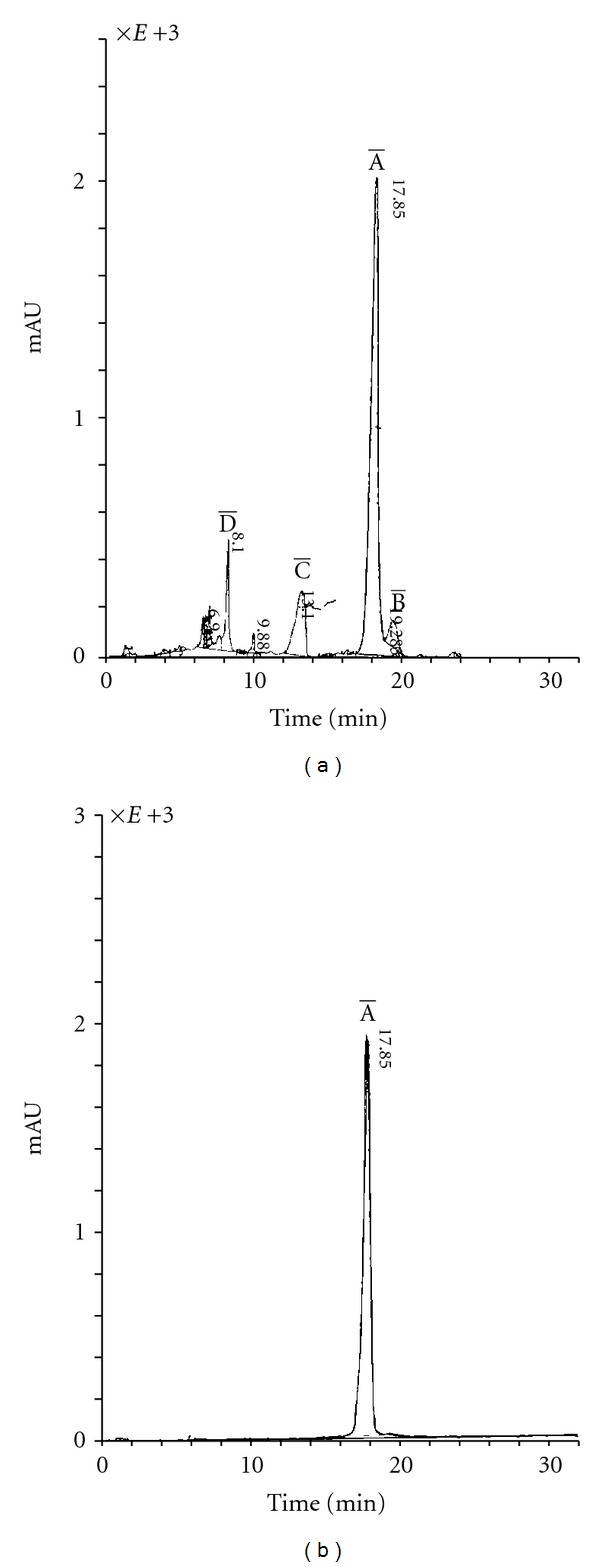
(a) *HPLC of antibiotic compound no. 70*. HPLC analysis was conducted on liquid chromatographer “Knauer” (Germany) with automated data processing system and spectrophotometric detector K = 2501. The chromatographic conditions used were as follows: flow rate, 1 mL/min; detection at wave length 260 and 300 nm; room temperature; loop, 20 *μ*L. Preparative isolation of components of the antibiotic compound A-70 was carried out through elution with a mixture of acetonitrile and water; the content of acetonitrile was gradually increased from 10 to 60%. The main component of the compound is the component with a retention time of 17.85 min. This component constitutes 72–74% of the compound, and was labeled as the component 70-A. Figure 2(a) shows the chromatogram of separation of the antibiotic compound no. 70. The component 70-B (retention time = 19.41 min) constitutes approximately 4% of total components. The components C and D are minor and together add up to less than 1%. The conditions for preparative isolation of individual components on chromatographer “Knauer” (Germany) were developed. The salt-free system acetonitrile-water in gradient elution mode from 10 to 60% of acetonitrile with detection at 300 nm met these optimal conditions. (b) *HPLC of the extracted and purified main component 70-A*. Conditions of HPLC analysis on liquid chromatographer “Knauer” (Germany) were developed for a more precise separation of the antibiotic compound no. 70 and determination of its components' ratio. By HPLC method, the chromatographically pure sample of the main component 70-A was isolated as shown in Figure 2(b).

According to the data of electrophoresis on paper, the antibiotic no. 70 was electroneutral. TLC on plates DC-Alufolien Kieselgel 60 “Merck” (Germany) with several solvent systems showed that the antibiotic no. 70 was a compound containing at least 4 biologically active components. In the system chloroform-benzene-methanol (30 : 20 : 7), the main component A has Rf = 0.65; the component B had an Rf = 0.35, while the components C and D were minor. Conditions of HPLC analysis on liquid chromatographer “Knauer” (Germany) were developed for a more precise separation of the antibiotic compound no. 70 and determination of its components' ratio (Figure 2(a)). Figure 2(a) shows the chromatogram of separation of the antibiotic compound no. 70.

As shown in Figure 2(a), the main component of the compound is the component with retention time of 17.85 min. This component constitutes 72–74% of the compound and was labeled as the component 70-A. The component 70-B (retention time = 19.41 min) constitutes approximately 4% of total components. The components C and D are minor and together add up to less than 1%. The conditions for preparative isolation of individual components on chromatographer “Knauer” (Germany) were developed. The salt-free system acetonitrile-water in gradient elution mode from 10 to 60% of acetonitrile with detection at 300 nm met these optimal conditions. By HPLC method the chromatographically pure sample of the main component 70-A was isolated (Figure 2(b)).  Table 2 summarizes certain physical-chemical characteristics of antibiotic 70-A.

The compiled data enabled the identification of 70-A through BNPD, a method developed by Béahdy (Hungary) [15]; the antibiotic compound no. 70-A was determined to exhibit characteristics closest to the antibiotic etamycin A [16–18], which belongs to the group of antibiotics peptolides-heteropeptidolactones [19, 20]. This group also includes other antibiotics such as etamycin, pristinamycin, and griseoviridin, as well as other antibiotics with high antibacterial activity and synergistic effect [20]. “Synercid,” the compound of semisynthetic antibiotics of this group, which includes quinupristin and dalfopristin, is highly active against a number of vancomycin-resistant bacteria and is currently being used in medicine [21, 22].

**Figure 3:**
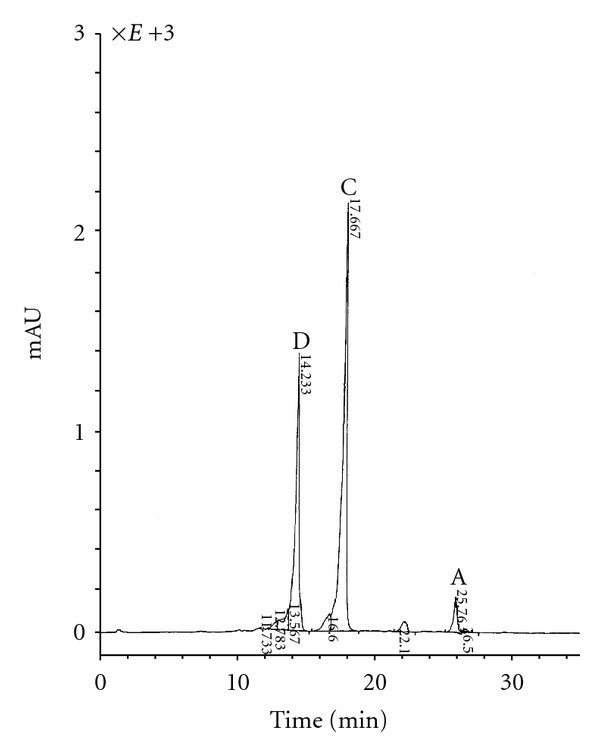
*Chromatogram of antibiotic compound no. 70, formed in medium A with soy flour*. The antibiotic compounds with a higher ratio of minor components and lower ratio of the main component A are formed on medium A with soy flour, in contrast to the medium containing yeast extract, whereby component A makes up to 74% of total compounds (as shown in Figure 2). Figure 3 shows the chromatogram of the main component of compound no. 70 formed in medium A with soy flour.

The direct comparison and the component 70-A and a sample of etamycin A by methods of TLC, HPLC, UV, MS-spectrometry showed that these compounds are identical. Thus, the antibiotic no. 70-A was identified as etamycin A with the same chemical structure represented (data not shown). As described above and previously [8, 14], fermentation conditions significantly influence antibiotic formation and component composition of the antibiotic compounds of antibiotic no. 70. The most efficient preparations are formed on medium B with yeast extract. The antibiotic compounds with a higher ratio of minor components and lower ratio of the main component A are formed on medium A with soy flour, in contrast to the medium containing yeast extract, whereby the component A makes up to 74% of the compound (Figure 3).

The new antibiotic components 70-C and 70-D were isolated by HPLC on “Knauer” (Germany), column 4 × 150 mm filled with sorbent “Chromasil A-100 C-18, 5 mcm” (BioChimMac, Moscow). Some of their associated characteristics are summarized in Table 3.

The components 70-C and 70-D have no analogues in BNPD, and quite possibly, have never been described before. The research of antibiotics 70-C and 70-D, which are novel components, is in progress. The findings of this study indicated that the antibiotic no. 70 has a significant potential application in medicine.

## 4. Discussions

One of the goals of this study was to determine the taxonomy of actinomycetes strain IMV-70, the producer of antibiotic compound no. 70. Preliminary screening and analysis by PCR method identified strain IMV-70 to be closely related to *Streptomyces niveoruber* and *Streptomyces thermodiastaticus, *but differs from them by cultural and morphological properties (data not shown). The optimal conditions for media and cultivation of IMV-70 were determined followed by development of methods for isolating and purifying the antibiotics no. 70. The latter study goal was performed to determine optimal methodologies and conditions which can be applied and adapted for large-scale preparations of the new antibiotic no. 70 and its purified components. The optimal media for biosynthesis of the antibiotic no. 70 were found to be the organic media with yeast and corn extracts, as well as soy, pea, and oat flour. The antibiotic was formed at the highest rate in the medium with yeast extract and peptone on the 4th–7th day of fermentation. We found in this study that the antibiotic no. 70 was active against gram-positive and gram-negative test organisms tested and was found to be active against virulent cultures of *Listeria monocytogenes, Erysipelothrix rhusiopathiae*, and* Y. pestis*. The overall antimicrobial activity was especially high against clinical cocci strains: staphylococci, streptococci, micrococci, enterococci, and aerococci with different types of drug resistance.

Research of the physical-chemical properties of the antibiotic compound shows that the antibiotic 70-A belongs to the heteropeptidolactone group, along with etamycin, pristinamycin, griseoviridin, and other antibiotics with high antibacterial and synergistic activity. The antimicrobial activities of antibiotic 70-A described in this study were consistent with reported anti-MRSA activities of naturally produced etamycin-isolated marine-derived actinomycetes [23].

In this study, we were able to determine the optimal growth conditions to cultivate actinomycetes producer strain IMV-70 and developed methods to isolate, purify, and accumulate preparations of the new antibiotic no. 70. The continuing research of the producer strain IMV-70 and its antibiotic compounds 70-A, 70-B and 70-C will enable us to discover the new nontoxic antibiotics, highly efficient against the clinical strains of MRSA and other dangerous infections.

## Figures and Tables

**Table:** (a)

Test organisms	Minimum inhibitory
	concentration, *μ*g/mL
*Comamonas terrigena *ATCC 8461	0.1
*Bacillus subtilis *ATCC 6633	0.25
*Staphylococcus aureus *FDA 209 P	0.05

**Table:** (b)

Microorganisms	Inhibition zone size (mm)
*S. haemolyticus *NMR 713	24
*S. haemolyticus *NMR 1626	22
*S. haemolyticus *NMR 1642	22
*S. haemolyticus* NMR 1742	24
*S. epidermidis* NMR 1656	22
*S. epidermidis* NMR 1694	21
*S. epidermidis* NMR *351 *	23
*S. epidermidis *NMR *793 *	22
*S. epidermidis *NMR *813 *	23
*S. haemolyticus *NMR *781 *	20
*S. lugdunensis *NMR *348 *	24
*Str. agalactiae *NMR *303 *	22
*Str. agalactiae *NMR *685 *	22
*Str. agalactiae *NMR 782	22
*Str. agalactiae *NMR 1002	25
*Str. oralis *NMR 1565	26
*Str. pneumoniae *NMR 1150	24
*Enterococcus hirae* NMR 459	19
*Enterococcus faecalis* NMR 425	16
*Enterococcus faecalis *NMR 118	16
*Enterococcus faecalis *NMR *670 *	17
*Enterococcus faecalis* NMR* 733 *	16

**Table 2:** Physical-chemical characteristics of antibiotic 70-A.

UV range (EtOH), *λ*_max,_ nm (E^1%^1 cm)	300 (84)

UV range (H_2_O), *λ*_max,_ nm (E^1%^1 cm)	340 (80)

HPLC on “Knauer” (Germany), column 4 × 150 mm filled with sorbent “Chromasil A-100 C-18, 5 mcm” (BioChimMac, Moscow), retention time, min.	17.85

Purity of the preparation according to HPLC data	95%

Molecular mass, method MS MALDI-TOF m/z	879 (M+H)^+^, 901 (M+Na)^+^, 917 (M+K)^+^

TLC, Kieselgel 60 (Merck) plates System: chloroform-benzene-methanol (30 : 20 : 7)	0.64

**Table 3:** Characteristics of certain minor components of antibiotic no. 70.

Components of antibiotic no. 70	UV-range, *λ*_max,_ nm, (C_2_H_5_OH)	Molecular mass, m/z, MS	HPLC, “Knauer”	
Components of antibiotic no. 70		MALDI-TOF method	Retention time, min.	Purity of the components, %
Component 70-C	210; **260.2;** 328 (bend)	917	17.53	97.06
Component 70-D	197; **248;** 302	622	14.11	93.0
